# Viral Diversity and Ecological Impact of DNA Viruses in Dominant Tick Species in China

**DOI:** 10.3390/microorganisms12081736

**Published:** 2024-08-22

**Authors:** Yueyang Yan, Zhangpeng Shi, Cunmin Wang, Zi Jin, Jigang Yin, Guan Zhu

**Affiliations:** 1 State Key Laboratory for Diagnosis and Treatment of Severe Zoonotic Infectious Diseases, Jilin University, Changchun 130062, China; yanyueyang@126.com (Y.Y.); cunminwang@163.com (C.W.); yinjg@jlu.edu.cn (J.Y.); 2 Institute of Zoonosis, Jilin University, Changchun 130062, China; rec3327559@gmail.com; 3 College of Veterinary Medicine, Jilin University, Changchun 130062, China; 4 Hangzhou Medical College, Hangzhou 310059, China; gloryjin@outlook.com

**Keywords:** ticks, viruses, metavirome, public health risks

## Abstract

Ticks are blood-feeding ectoparasites that also transmit various pathogens, posing severe risks to human and animal health. DNA viruses play a crucial role in the microbial ecology of ticks, but their distribution and ecological significance remain largely undetermined. Here, we assembled an extensive catalog encompassing 4320 viral operational taxonomic units (vOTUs) from six main dominant tick species in China, of which 94.8% have not been found in any other environment. To bridge the knowledge gap in tick DNA virus research and provide a crucial resource platform, we developed the Tick DNA Virus Database. This database includes the vOTUs that are known to cause diseases. Most of the predicted vOTUs are associated with dominant bacterial and archaeal phyla. We identified 105 virus-encoded putative auxiliary metabolic genes (AMGs) that are involved in host metabolism and environmental adaptation, potentially influencing ticks through both top-down and bottom-up mechanisms. The identification of microbial communities and antibiotic resistance in wild tick species suggests that wild ticks are reservoirs of antibiotic resistance and potential spreaders of antibiotic resistance. These findings reveal the potential role of tick viruses in ecosystems, highlighting the importance of monitoring tick microbiomes to address global public health challenges.

## 1. Introduction

Ticks are blood-feeding ectoparasites that also transmit vector-borne diseases to humans and/or animals [1]. More than 100,000 cases of tick-borne diseases are reported around world annually [2]. Tick-transmitted pathogens include protozoan parasites, bacteria and viruses [3,4]. Ticks might harbor a diverse group of viruses, many of them co-evolve with the hosts, forming a complex relationship that influences the physiological functions, behavior, and interactions of ticks with other microorganisms [2,5,6]. They may trigger various immune responses in ticks and potentially affect the tick populations [1,7]. Therefore, understanding the whole viral compositions in ticks would provide not only important information on the risks of tick-transmitted viral pathogens to humans and animals but also opportunities to investigate the complex tick–virus interactions.

The development of viral metagenomics has revolutionized the study of tick viruses, allowing the impartial capture of the viral communities in ticks [7]. For instance, a meta-transcriptomic study by Ye and colleagues on longhorned ticks in Shandong Province, China, identified 48 viral species and 508 RNA viruses, 22 of which were previously unreported [8]. Another study by Zakham and colleagues used a metagenomic approach to characterize the virome of ticks infesting dromedary camels (*Camelus dromedarius*) in the Mecca Province, Saudi Arabia [9], detecting various tick-specific viruses including *Phenuiviridae*, *Iflaviridae*, *Chuviridae*, *Totiviridae*, and *Flaviviridae*. Nonetheless, many studies on tick viromes have remained primarily focused on describing the virome compositions [7,10,11]. The potential impact of tick viruses on host ecosystems and their risks to animal and human health remain underexplored.

In this study, we analyzed 3.4 TB of tick metagenomic data from public databases, derived from 622 tick samples collected from various regions in China, and constructed 4320 DNA viral genomes. We also developed a Tick DNA Virus Database (http://39.101.72.186:8081/ accessed on 1 August 2024). In addition to studying the viral compositions in ticks, we also attempted to identify new viral species and viral pathogens, with potential risks to human or animal health, and the bacterial hosts for the viruses. We also examined auxiliary metabolic genes and antibiotic resistance genes (ARGs) carried by the viral genomes to infer the ecological roles of viruses. These genomes expand our knowledge of DNA virus diversity in the tick microbiomes and understanding of host–virus interactions, providing a new foundation for assessing the risks of tick-borne viral transmission to humans and animals.

## 2. Results

### 2.1. A Genomic Catalogue of DNA Viruses from the Tick

**Figure 1:**
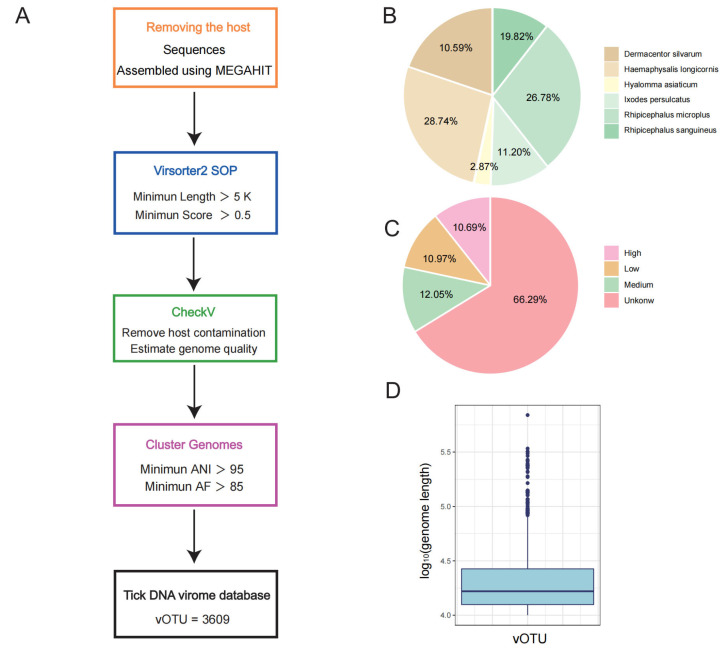
Recovery of tick DNA virus genomes. (**A**) Overview of the viral genome discovery workflow. (**B**) Numbers of viral genomes from different tick species. (**C**) Estimated genome quality (high, completeness > 90%; medium, completeness 50–90%; low, completeness < 50%; unknown, completeness uncertain). (**D**) Genome size statistics. vOTU, viral genome taxonomic operational unit.

We collected raw metagenomic reads from 622 tick samples originated from 27 provinces, municipalities, and autonomous regions across Mainland China from a public database (Figure 1A) [12]. These metagenomic data encompass the main dominant tick species in China, including *Ixodes sinensis*, *Ixodes persulcatus*, *Haemaphysalis longicornis*, *Dermacentor silvarum*, *Hyalomma asiaticum*, *Rhipicephalus sanguineus*, and *Rhipicephalus microplus* (Figure 1B). Based on this, we have developed large niche-specific tick DNA virome databases.

We processed 3.2 TB of metagenomic data and assembled them after removing reads derived from the tick hosts. Following the recommendations of a recent viromic benchmarking paper and stringent criteria [13], we identified 6024 putative viral genomes, each greater than 5 kilobases in length. The completeness of the assembled viral metagenomes or genome fragments were assessed using CheckV, which were categorized into four quality levels (Figure 1C), including high (8% of the assembled viral genomes, >90% completeness), medium (10%, 50–90% completeness), and low quality (9%, 0–50% completeness). The remaining genomes were undetermined. The completeness and quality of tick DNA genomes, or viral genome operational taxonomic units (vOTUs), might be underestimated because CheckV relies on databases primarily derived from other ecosystems.

To quantify the diversity of genomes in the tick DNA virome catalog [14], we first identified species-level vOTUs using the MIUViG recommended criteria as follows: 95% average nucleotide identity (ANI) over 85% of the length of the shorter sequence, with an average length of 23 kb (Figure 1D). Similar to human intestinal viruses [1,15], we observed diversity within the individual samples and shared viral species across the different samples. Overall, we identified over 3000 vOTUs, in which 1168 were present in at least two samples. To facilitate further research and applications, we constructed a tick DNA virome database, available for download at http://39.101.72.186:8081/ accessed on 1 August 2024.

### 2.2. Tick DNA Viruses Are Highly Diverse and Novel

**Figure 2:**
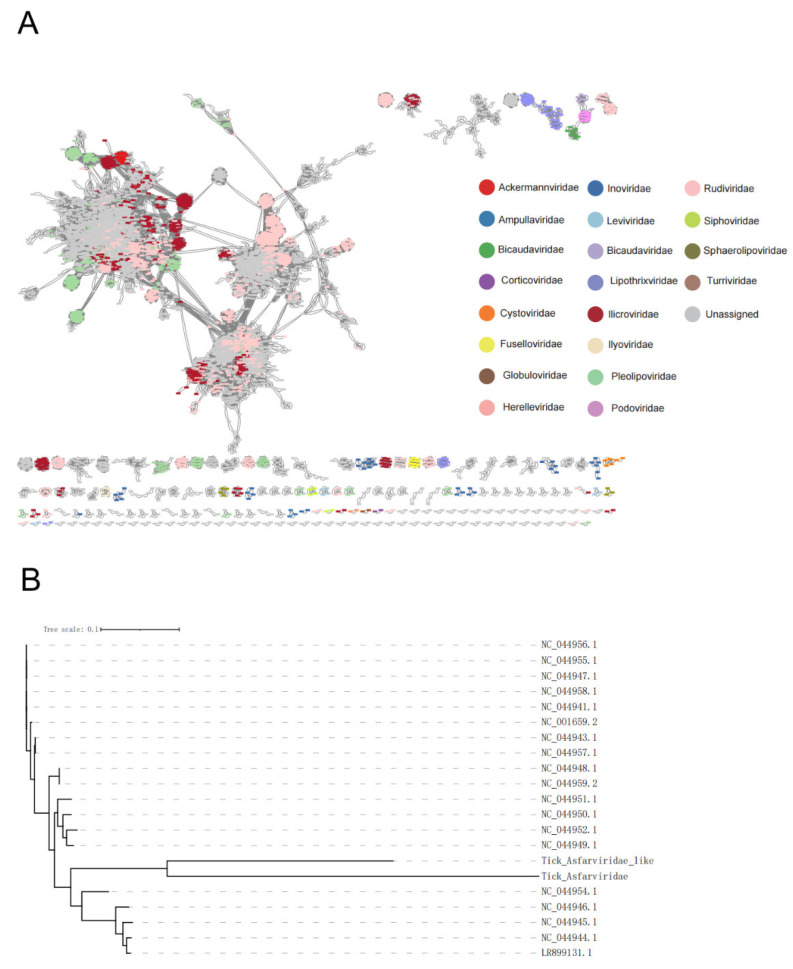
Classification of Tick DNA viral metagenomes. (**A**) vOTU gene-sharing network obtained from vConTACT2, visualized using Cytoscape. Viral genomes are colored according to their family assignment. (**B**) Phylogeny of African swine fever virus and its homologues. For each sequence, the accession number and virus name/strain are provided.

Based on the gene-sharing network constructed using vConTACT2, this study analyzed the uniqueness of tick viral vOTUs by comparing them with viral sequences from the IMG/VR 3.0 database that contained over 2 million cultured and uncultured viral sequences (Figure 2A). This weighted network assigned sequences to viral clusters mostly at the genus level. Only ~12% of the viral vOTUs clustered with taxonomically known genomes from the Viral RefSeq, indicating that most tick vOTUs were unique in comparison to the viruses from other ecosystems. These unique vOTUs likely represent new viral lineages.

In 2021, the International Committee on Taxonomy of Viruses (ICTV) updated the virus classification criteria. Consequently, this study employed a semi-supervised machine learning model, PhaGCN [16], based on a graph convolutional network, to automatically update the database and annotate the viral genomes at the family level according to the new ICTV system. Among 53.41% of the successfully annotated vOTUs, 125 families were identified. The majority of vOTUs were assigned to families such as *Peduoviridae*, *Hendrixvirinae*, *Azeredovirinae*, and *Peduovirida*. The study also identified viral families associated with vertebrate diseases, including *Herpesviridae*, *Adenoviridae*, *Asfarviridae*, and *Iridoviridae*. Although these pathogens were relatively few in number, they have the potential to cause common and serious diseases in livestock.

A notable pathogen present in the tick viromes was the African Swine Fever Virus (ASFV) in the Family *Asfarviridae*, confirming that ticks were the vector, or one of the vectors, for the transmission of ASFV. ASFV is one of the most destructive pathogens affecting pigs [17], causing severe economic losses in the swine industry due to its high mortality rates and the absence of effective vaccines or treatments. Taxonomically, ASFV is the sole member of the genus and family, and the only known DNA arbovirus. Phylogenetic analysis clustered the ASFV genomes from the tick viromes with ASFV or ASFV-like genomes reported in the GenBank database (Figure 2B). This indicates that the tick-associated ASFV and ASFV-like viruses are genetically similar, suggesting a potentially shared evolutionary path. The identification and classification of these ASFV-like viruses expand our understanding of the diversity within the *Asfarviridae* family.

### 2.3. Tick DNA Viruses Have a Narrow Bacterial Host Range

**Figure 3:**
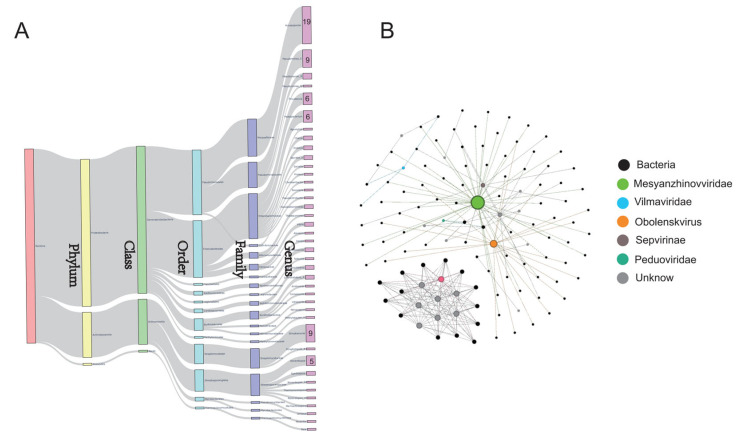
The bacterial host range of tick DNA viruses. (**A**) The Sankey diagram illustrates the taxonomic classification of bacterial hosts identified in tick samples, showing a detailed breakdown of the relationships from the phylum to the genus level. (**B**) The network visualization shows the relationships between the viruses and the host bacteria. Nodes represent individual bacteria and viruses, and edges indicate that a virus can infect certain bacteria. The colors of the nodes represent different viral families or unknown classifications. If a vOTU node is connected to different host nodes, it indicates that the vOTU is predicted to link to different host categories, suggesting a potentially broad host range.

Understanding host predictions is crucial for comprehending the potential roles of viruses within ecosystems. Our analysis predicted that tick DNA viruses mainly infect 99 bacterial species in three dominant bacterial phyla, including Actinobacteriota, Firmicutes, and Proteobacteria (Figure 3A). The predominant genera included *Acinetobacter* (e.g., *A. proteolyticus* and *A. ursingii*), *Streptomyces* (e.g., *S. silvensis* and *S. globosus*), *Pseudomonas* (e.g., *Pseudomonas_E parafulva*), *Providencia* (e.g., *P. burhodogranariea*), and *Pectobacterium* (e.g., *P. brasiliense*). The prediction indicates a degree of host specificity, suggesting that these viruses have evolved mechanisms to interact effectively with these particular bacterial groups, likely as a result of co-evolutionary relationships.

Through comparison of the CRISPR spacer regions, we found that 0.5% of the 4320 tick viral vOTUs were predicted to have putative hosts. This method enabled us to infer which bacteria had been previously infected by these viruses, as the CRISPR spacer regions serve as a genetic record of past viral encounters. Consistent with previous observations [18], the majority of these vOTUs were predicted to have a narrow host range, infecting specific bacterial species or genera. However, some vOTUs exhibited a broader host range, capable of infecting bacteria across multiple phyla, indicating a higher level of adaptability and ecological versatility (Figure 3B). Notably, the *Mesyanzhinovviridae* family had the broadest host range, showing the potential to infect bacterial hosts from different phyla.

### 2.4. Virus Auxiliary Metabolic Genes Help Ticks to Adapt to a Blood-Feeding Lifestyle

**Figure 4:**
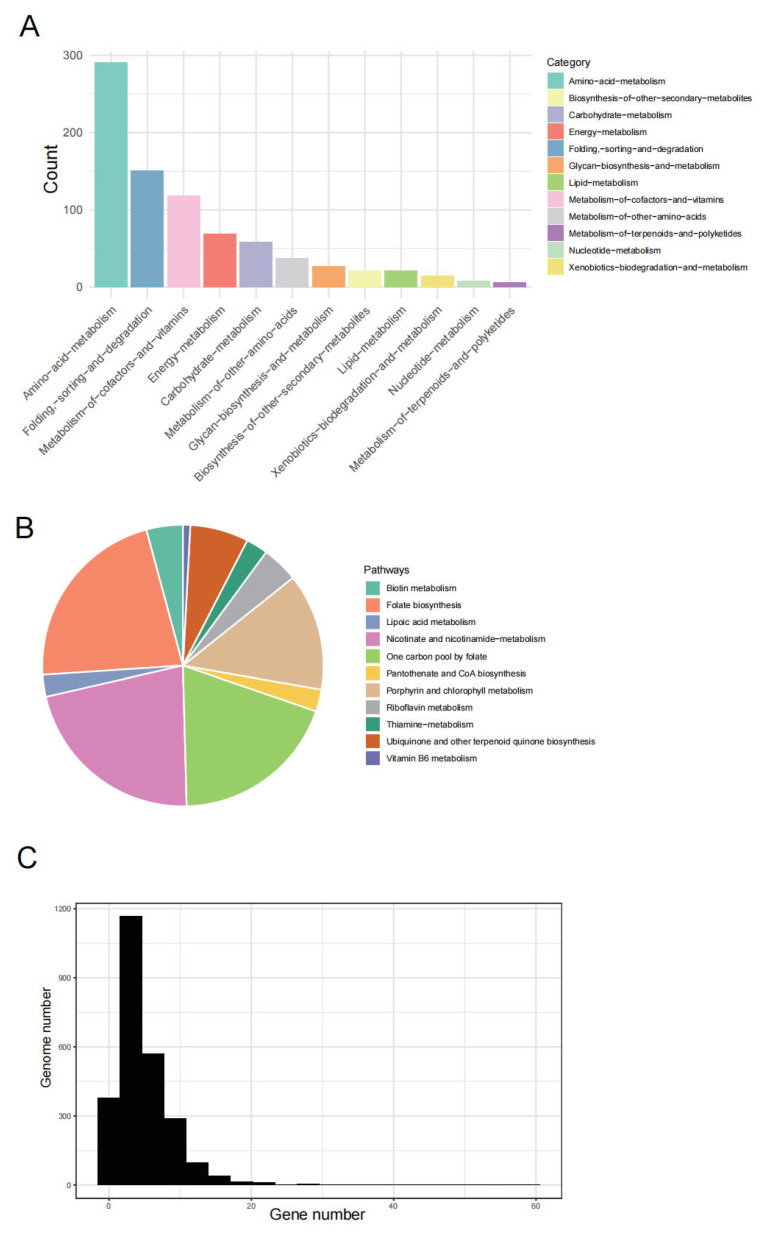
Auxiliary metabolic genes (AMGs) carried by viruses. (**A**) KEGG functional categories of protein-coding viral AMGs identified in all viral genomes. (**B**) Classification of AMGs within the metabolism of cofactors and vitamins pathways. (**C**) Numbers of viral genomes containing AMGs.

To understand how viruses impact ticks, we studied auxiliary metabolic genes (AMGs) encoded by vOTUs. Based on VIBRANT annotations, these AMGs represent 12 distinct categories, primarily involved in various metabolic processes, including amino acid metabolism and degradation, cofactor and vitamin metabolism, and energy metabolism (Figure 4A). The most prevalent AMG encodes DNA methyltransferase (DNMT1), present in more than 227 vOTUs. DNMT1 is part of the viral immune evasion machinery by methylating bacteriophage/viral DNA to prevent the attack by the restriction–modification (RM) systems in the bacterial hosts. Its high prevalence is consistent with observations in marine viromes [19] and rumen environments [20]. This AMG likely represents a widespread anti-defense mechanism employed by viruses.

Genomes that have established long-term associations with the hosts tend to lose genes that are not under selective pressure, while retaining genes beneficial to themselves and/or their hosts. This genome “optimization” was also corroborated in our study of tick vOTUs. We found that tick vOTUs encode enzymes related to cofactor and vitamin metabolism, including key enzymes involved in folate biosynthesis, nicotinate, and nicotinamide and biotin metabolism (Figure 4B).

These findings suggest that viruses might influence host microbiomes, potentially optimizing bacterial host metabolism in adaptation to their unique blood-feeding niche. Particularly under nutrient-scarce conditions, these viruses may confer additional metabolic advantages to ticks. Further analysis revealed that 51.8% of vOTUs carry more than one AMG (Figure 4C), indicating that these diverse tick viruses are functionally complex and broad. The high proportion of AMG carriage suggests that viruses exert significant influence on the metabolic activities of their host microbiomes.

### 2.5. Tick Viruses May Facilitate the Transmission of ARGs across Phylogenetic Boundaries

**Figure 5:**
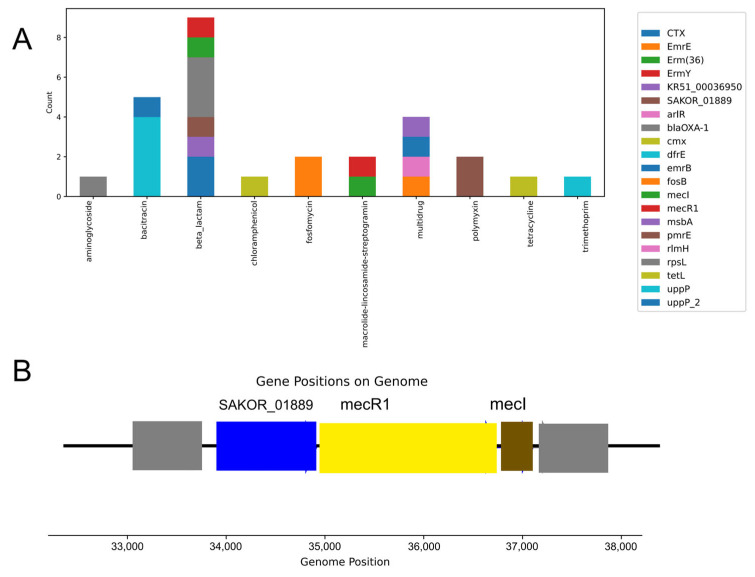
Antibiotic resistance genes (ARGs) carried by viruses from ticks. (**A**) Categories of identified ARGs. (**B**) Genomes of representative viral contigs carrying ARGs.

To investigate the antibiotic resistance genes (ARGs) in tick-borne viruses, we annotated them using the established ARG databases [21]. Consistent with previous observations on viruses, the scarcity of viral ARGs confirms the low prevalence of ARGs in the phage genomes. In the tick viruses, 29 ARGs were identified from the 4320 viral genomes that were categorized into 10 resistance types. The predominant ARG types include beta-lactam, bacitracin, multidrug, and macrolide-lincosamide-streptogramin (Figure 5A).

These ARGs are categorized based on their resistance mechanisms into antibiotic inactivation, cellular protection, efflux pumps, and unknown mechanisms. These resistance gene types confer resistance to major classes of antibiotics commonly employed in clinical and agricultural settings. While most viruses carry only one ARG, some compact viral gene structures harbor multiple ARG subtypes. A representative example is a virus carrying the beta-lactam ARGs mecI, mecR1, and SAKOR_01889 (Figure 5B). This compact gene structure suggests that these genes may work together to provide stronger resistance. As arthropod vectors, ticks can disseminate viruses and associated ARGs between animal species through their movement and multi-host blood-feeding lifestyle.

## 3. Discussion

Global warming impacts the Earth, altering climate conditions in many ecosystems and consequently changing the habitat distribution of animal and human disease vectors [22]. As temperatures rise, the habitat range of ticks expands, facilitating the spread of various pathogenic microorganisms. Although ticks are important vectors, the risk of most tick-associated viruses to humans and animals remains largely uncertain. In this study, we identified over 3000 vOTUs and constructed a database of tick DNA viruses, including diverse and previously uncharacterized viral groups. This significantly expanded the current knowledge of tick-associated viruses. Our analyses on the viral genome compositions also provided new insights into tick host and virus interactions.

Epidemiological information on tick-borne diseases is crucial for controlling these diseases. Our understanding of tick-borne RNA viruses is relatively comprehensive, but research on the diversity of tick-borne viruses (TBVs) has largely been neglected [23]. Our study found that tick-borne DNA viruses not only exhibit genetic diversity but also include various potentially pathogenic viruses. This study represents the first instance of *Asfarviridae* genomes being assembled using metagenomic methods from ticks, including both *Asfarviridae* and *Asfarviridae*-like viruses. ASFV is the only known DNA arbovirus, capable of being transmitted by ticks across hosts, genders, and through transovarial transmission, and is one of the most devastating swine pathogens [24]. Viruses from the *Asfarviridae* family have been detected in diverse tick populations worldwide. For example, *Asfarviridae* viruses have been found in *Ornithodoros moubata* in France, *O. moubata* in Germany, *O. porcinus* in Kenya, *O. erraticus* in Portugal, and *Rhipicephalus microplus* in China [24]. Given the lack of effective treatments and vaccines, a deeper understanding of ASFV epidemiology is helpful for developing strategies to limit the spread of the virus [25]. The high-risk vectors identified in this study can be used to assess the risk of ASFV transmission, guide targeted surveillance and control strategies, and facilitate proactive responses to ASFV invasion events.

Compared to viruses in the human intestines [15], animal rumens [19], and urban environments [26], tick viruses exhibit a narrower host range. This singular ecological niche implies host range specialization [27]. As ectoparasites, ticks have a specialized lifestyle, feeding exclusively on the blood of vertebrate hosts. This specialization exerts distinct evolutionary pressures on the viruses they carry [25,28], driving the development of a more restricted host range. Despite this narrow host range, a commonality between tick viruses and other environmental viruses is their ability to infect bacterial hosts across different phyla. This suggests that tick viruses could potentially mediate genetic exchange across phylum boundaries, promoting microbial adaptation and evolution [17]. Genetic exchanges could lead to new traits and abilities in microbial populations, enhancing their adaptation to the changing environments. Therefore, tick viruses not only play an important role within their specialized ecological niche but also have a profound impact on broader microbial ecosystems.

The viromes might influence hosts in a top-down manner, particularly through the action of virus-encoded AMGs. In ticks, viruses might impact the metabolism of their bacterial hosts by manipulating nutrient acquisition and metabolic pathways [29]. For example, tick viral genomes contain genes encoding enzymes to synthesize B vitamins, riboflavin, biotin, and folate. Vertebrate blood, which is deficient in certain vitamins, makes it necessary for the ticks to maintain symbionts capable of supplying these essential nutrients. Tick viruses do not possess complete vitamin synthesis pathways, but they encode key enzymes to supplement the vitamin metabolism [30]. These viral AMGs compensate the metabolic needs of the host, providing an additional survival advantage in nutrient-poor environments.

In the context of the One Health framework, the presence of tick viral ARGs indicates that viruses may help their hosts survive in the environments with antibiotics introduced by human activities or by certain antibiotic-producing microbes. Most of these ARGs are related to antibiotics used in humans and animals, including those considered critically important for human medicine by the World Health Organization (WHO). Ticks are significant reservoirs for pathogens of humans and/or animals [31]. Monitoring the tick microbiome, including the virome, is helpful for understanding the potential risk of tick-transmitted bacterial and viral diseases of humans and animals.

## 4. Conclusions

This study assembled more than 3000 vOTUs of tick viruses and revealed a diversity of novel viruses from ticks that were previously uncharacterized viral groups. These data provide some new insights to the understanding of tick host–virus interactions and unveil the potential impact of tick viruses on the associated microbial communities. This study also detected a number of potential human and animal viral pathogens (e.g., ASFV), highlighting the importance of monitoring tick viromes in the risk assessment of tick-transmitted viral diseases. The survey of tick viromes also provides information to assist in the development of biocontrol strategies and the management of vector-borne diseases.

## 5. Method

### 5.1. Data Collection

This study analyzed datasets derived from a metagenomics study of six species of ixodid ticks collected between 2017 and 2019 in China, including *Dermacentor silvarum*, *Haemaphysalis longicornis*, *Hyalomma asiaticum*, *Ixodes persulcatus*, *Rhipicephalus microplus*, and *Rhipicephalus sanguineus* (BioProject: PRJNA633311; BioSample: SAMN14941059). The collection and DNA sequencing procedures are described in detail elsewhere [12]. In brief, from November 2017 to January 2019, researchers in Mainland China collected ticks using a standard 1 m^2^ flannel flag or directly from domestic and wild animals, recording the latitude and longitude of each site. Wild-caught ticks were surface-sterilized, and libraries were sequenced on an Illumina NovaSeq platform, generating paired-end reads.

### 5.2. Assembly and Identification of Viral Genomes

Quality control was performed using fastp [32] with default parameters. Sequences were then aligned to the tick genome using BWA [33], and the host sequences were removed using samtools. Each tick sample’s quality-controlled reads were assembled separately using MEGAHIT [34] with default parameters. Contigs shorter than 5000 bp were discarded. VirSorter2 [35] (option: --min-score 0.5) was used to recover provisional viral contigs >5 kb from metasgenome assemblies. Viral contigs larger than 5 kb were selected because a relatively high false positive rate has been exhibited by the current bioinformatics tools when identifying viral contigs smaller than 5 kb.

These contigs were then input into CheckV [36], an automated pipeline for identifying closed viral genomes, estimating the completeness of genome fragments, and removing flanking host regions from integrated proviruses. Viral contigs were clustered into vOTUs based on 95% average nucleotide identity (ANI) over 85% of the shortest contig [16]. All genes in the identified viral genomes were predicted using Prodigal [37].

### 5.3. Virus–Host Prediction

We collected all prokaryotic genomes from The Genome Taxonomy Database [38] (GTDB: https://gtdb.ecogenomic.org, accessed on 1 August 2024.). CRISPR-Cas protospacers were identified and extracted from MAG sequences using the CRISPR Recognition Tool (CRT). BLASTn was then used to compare these MAG protospacer sequences with those in vMAGs. Matches were retained only if they were 100% identical or contained ≤1 bp mismatch with an e-value of ≤1 × 10^−5^.

### 5.4. Taxonomic Classification of All vOTUs

To classify the viral operational taxonomic units (vOTUs) larger than 5 kb, we employed protein–protein similarity analysis using Diamond. This analysis was based on gene-sharing networks constructed with vConTACT2 [39], using the NCBI RefSeq Viral database as the reference genome. Following the protocol published on protocols.io (https://www.protocols.io/view/applying-vcontact-to-viral-sequences-and-visualizi-x5xfq7n accessed on 1 August 2024), vConTACT2 was applied, and the resulting gene-sharing network was visualized in Cytoscape [40]. Given the revisions in viral taxonomy by the International Committee on Taxonomy of Viruses (ICTV) in 2021, further annotation of vOTUs was performed using PhaGCN [15], which utilizes the latest ICTV classification. PhaGCN is a semi-supervised machine learning model based on graph convolution networks, optimizing learning from DNA sequence-constructed knowledge graphs.

### 5.5. Functional Annotation

Gene annotation was performed using VIBRANT [41], which searches across various databases, including the Kyoto Encyclopedia of Genes and Genomes (KEGG) [42], PFAM [43], and VOG [44] to identify viral-like proteins. If the best HMM hit corresponded to the KEGG database and the annotation was part of a metabolic pathway, the gene was classified as an Auxiliary Metabolic Gene (AMG).

## Data Availability

The viral genomes can be accessed and downloaded without any restriction at https://figshare.com/articles/online_resource/tick-DNAviruses/26394661 accessed on 1 August 2024.
